# Dataset on the effect of heat-treatment temperature on the cycle and rate properties of MoSe_2_/C composite nanofibers as anodes for sodium ion batteries

**DOI:** 10.1016/j.dib.2019.104018

**Published:** 2019-05-18

**Authors:** Sun Young Jeong, Jung Sang Cho

**Affiliations:** Department of Engineering Chemistry, Chungbuk National University, Chungbuk, 361-763, Republic of Korea

**Keywords:** Heat-treatment temperature, MoSe_2_, Nanofibers, Anodes, Na ion batteries

## Abstract

The data presented in this article are related to the research article entitled “Multi-channel-contained few-layered MoSe_2_ nanosheet/N-doped carbon hybrid nanofibers prepared using diethylenetriamine as anodes for high-performance sodium-ion batteries” Jeong et al., 2019. The data presented in this manuscript showed the effect of the selenization temperature of the as-spun fibers on the cycle and rate properties as anodes for sodium ion batteries. Each morphology, phase, and the resulting cycle and rate properties of the MoSe_2_/C composite nanofibers obtained after various selenization temperatures were investigated.

**Table:** Specifications table

Subject area	*Chemistry*
More specific subject area	*Inorganic chemistry*
Type of data	*Figures*
How data was acquired	*FE-SEM (JEOL, JSM-6060), XRD(X'Pert PRO MPD, PANalytical), cycle and rate properties (2032-type coin cell)*
Data format	*Raw, analyzed data*
Experimental factors	*Selenization temperature*
Experimental features	*Morphology, crystallite size of MoSe*_*2*_*, and cycle property*
Data source location	*Cheongju, Chungbuk, Republic of Korea*
Data accessibility	*Data included in this article*
Related research article	*S. Y. Jeong, S. Ghosh, J.-K. Kim, D.-W. Kang, S. M. Jeong, Y. C. Kang, J. S. Cho, Multi-Channel-Contained Few-Layered MoSe*_*2*_*Nanosheet/N-doped Carbon Hybrid Nanofibers Prepared Using Diethylenetriamine as Anodes for High-Performance Sodium-Ion Batteries “in press”*[1]

**Table:** 

**Value of the data** • These data provide the appropriate selenization temperature to obtain MoSe_2_/C composite nanofibers with excellent sodium ion storage properties. • To understand the effect of selenization temperature on the obtained nanofiber morphology, crystallite size of MoSe_2_. • These data could be applied to control crystallite size of other metal compounds.

## 1 Data

**Fig. 1:**
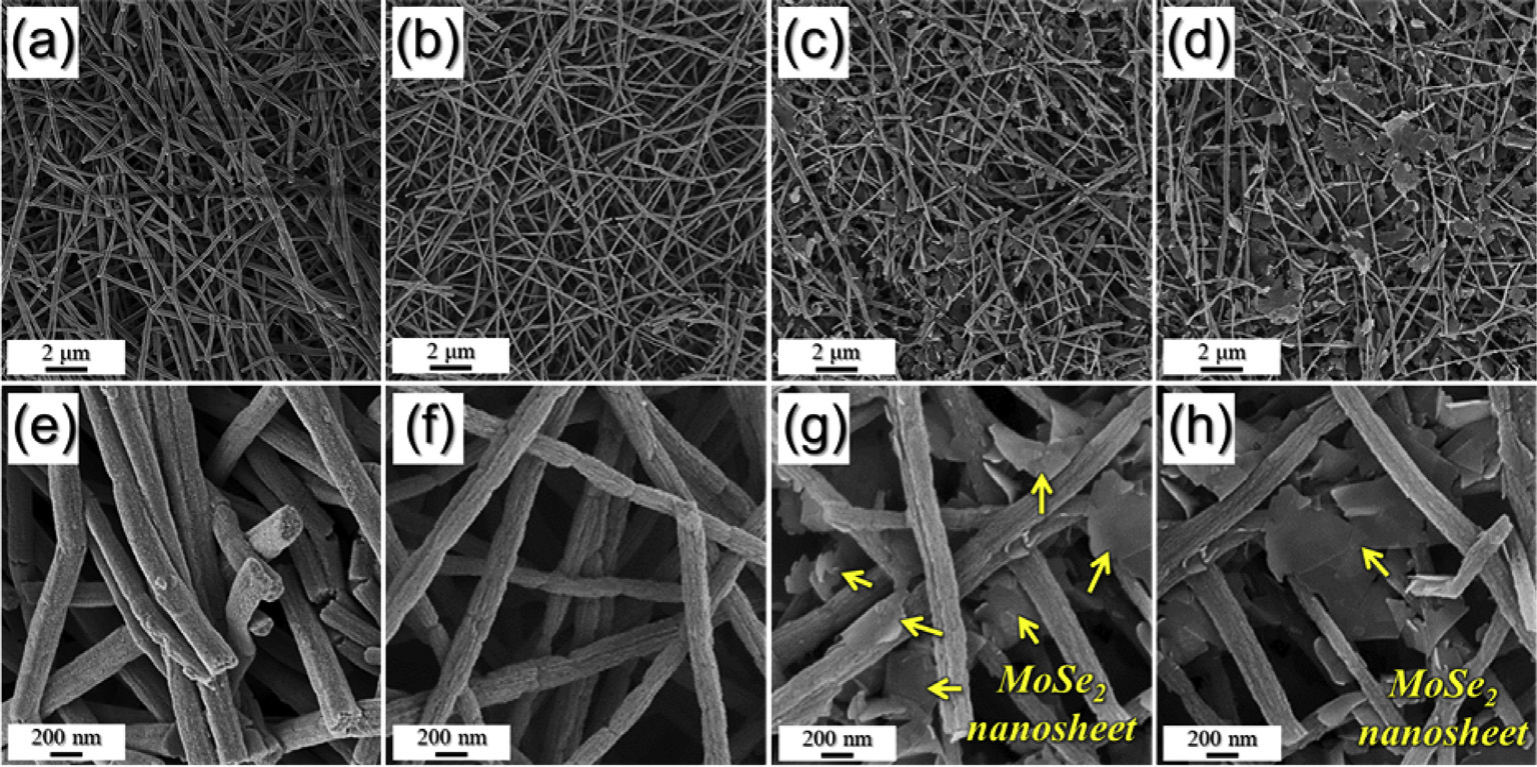
FE-SEM images of the samples: (a,e) Sel200, (b,f) Sel400, (c,g) Sel600, and (d,h) Sel800.

**Fig. 2:**
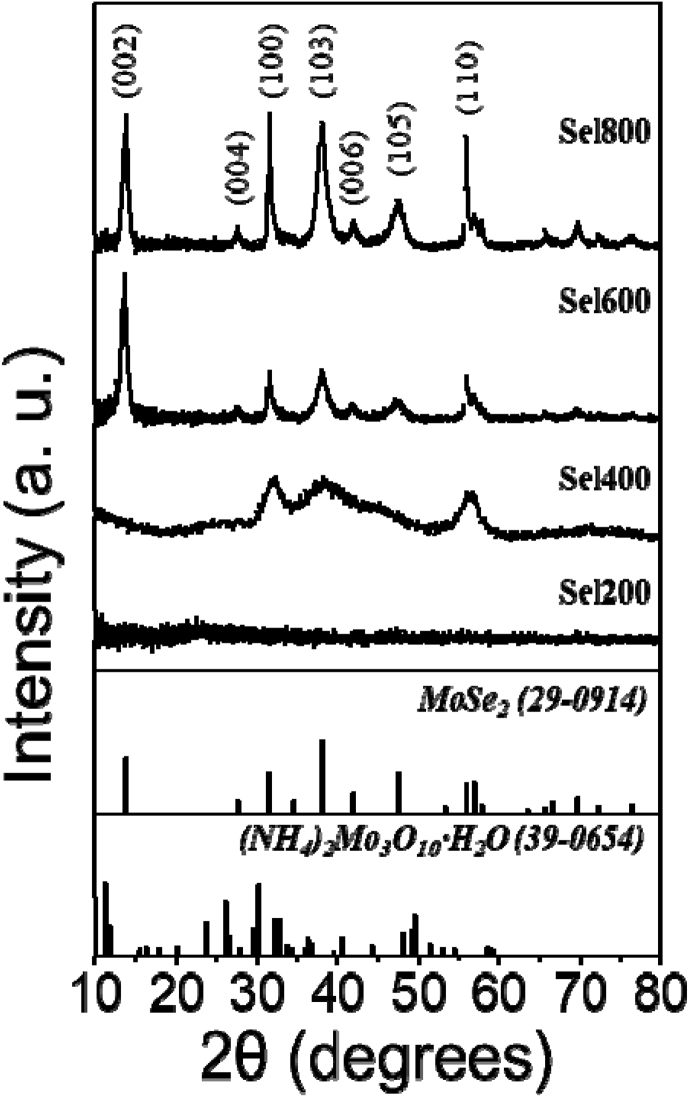
XRD patterns of the samples obtained at various selenization temperatures.

**Fig. 3:**
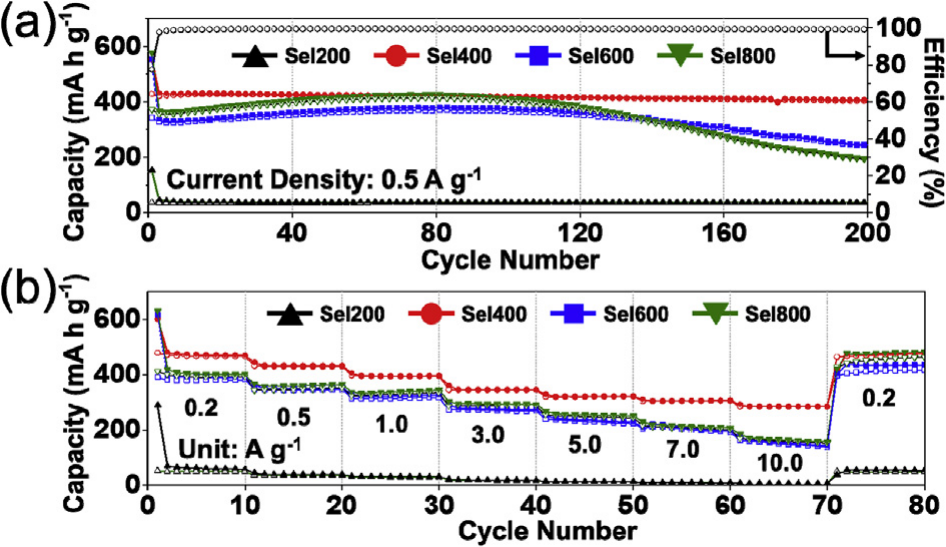
Electrochemical properties of Sel200, Sel400, Sel600, and Sel800: (a) cycle performances and (b) rate performances.

The data exhibited in this manuscript include that showing the effect of selenization temperature on the morphology and crystallite size of the MoSe_2_ of the multi-channel contained MoSe_2_/C composite nanofiber. Fig. 1 shows nanofiber morphologies obtained after selenization with various temperatures. Fig. 2 shows the XRD patterns of nanofibers obtained after selenization with various temperatures. Fig. 3 shows the electrochemical properties of the samples obtained by half-cell electrochemical test.

## 2 Experimental design, materials, and methods

Experimental details are written in the reference [1]. Briefly, ammonium molybdate, polyvinylpyrrolidone, and diethylenetriamine were added to distilled water to prepare the spinning solution. The solution was then electrospun and stabilized at 150 °C. To understand the effect of the selenization temperature on the morphology of the nanofibers and their crystallite size of MoSe_2_ in the composites, nanofibers were undergone selenization at 200 °C, 400 °C, 600 °C, and 800 °C, respectively. These samples were denoted Sel200, Sel400, Sel600, and Sel800, respectively. Morphologies of the samples were observed by FE-SEM and exhibited in Fig. 1. All samples showed one-dimensional nanofiber morphology. However, as the selenization temperature increased above 600 °C, sheet-typed MoSe_2_ were also generated along with nanofibers.

The crystallite size of MoSe_2_ in the composites obtained at various selenization temperatures were shown in Fig. 2. Sel200 showed the amorphous-like phase. However, above selenization temperature of 400 °C, hexagonal MoSe_2_ phase was formed. The mean sizes of nanocrystallites comprising Sel400, Sel600, and Sel800, calculated by Scherrer's equation to the (100) MoSe_2_ peak were 3, 12, and 17 nm, respectively.

The cycle properties of the samples at a current density of 0.5 A g^−1^ were shown in Fig. 3. The initial discharge capacities of Sel200, Sel400, Sel600, and Sel800 were 154, 553, 555, and 577 mA h g^−1^, respectively, and their Coulombic efficiencies were 23, 77, 62, and 65%, respectively. The discharge capacities of Sel200, Sel400, Sel600, and Sel800 after 200 cycles were 36, 406, 247, and 193 mA h g^−1^, respectively. The rate performances of the samples at various current densities between 0.2 and 10.0 A g^−1^ are shown Fig. 3b. Sel400 showed the best rate property among samples. The final discharge capacities of Sel400 at 0.2, 0.5, 1.0, 3.0, 5.0, 7.0, and 10.0 A g^−1^ were 470, 431, 397, 345, 322, 307, and 285 mA h g^−1^, respectively. The discharge capacity recovered to 478 mA h g^−1^ when current density back to 0.2 A g^−1^.
